# The Association between Body Mass Index and Mortality in Incident Dialysis Patients

**DOI:** 10.1371/journal.pone.0114897

**Published:** 2014-12-16

**Authors:** Sunil V. Badve, Sanjoy K. Paul, Kerenaftali Klein, Philip A. Clayton, Carmel M. Hawley, Fiona G. Brown, Neil Boudville, Kevan R. Polkinghorne, Stephen P. McDonald, David W. Johnson

**Affiliations:** 1 Department of Nephrology, Princess Alexandra Hospital, Brisbane, Queensland, Australia; 2 Clinical Trials & Biostatistics Unit, QIMR Berghofer Medical Research Institute, Brisbane, Queensland, Australia; 3 Department of Renal Medicine, Royal Prince Alfred Hospital, Sydney, New South Wales, Australia; 4 Department of Nephrology & Medicine, Monash Medical Centre and Monash University, Melbourne, Victoria, Australia; 5 School of Medicine and Pharmacology, University of Western Australia, Perth, Western Australia, Australia; 6 Australia and New Zealand Dialysis and Transplant Registry, University of Adelaide at Central Northern Adelaide Renal & Transplant Services, Adelaide, South Australia, Australia; University of Washington, United States of America

## Abstract

**Objectives:**

To study the body mass index (BMI) trajectory in patients with incident end-stage kidney disease and its association with all-cause mortality.

**Methods:**

This longitudinal cohort study included 17022 adult patients commencing hemodialysis [HD] (n = 10860) or peritoneal dialysis [PD] (n = 6162) between 2001 and 2008 and had ≥6-month follow-up and ≥2 weight measurements, using the Australia and New Zealand Dialysis and Transplant Registry data. The association of time-varying BMI with all-cause mortality was explored using multivariate Cox regression models.

**Results:**

The median follow-up was 2.3 years. There was a non-linear change in the mean BMI (kg/m^2^) over time, with an initial decrease from 27.6 (95% confidence interval [CI]: 27.5, 27.7) to 26.7 (95% CI: 26.6, 26.9) at 3-month, followed by increments to 27.1 (95% CI: 27, 27.2) at 1-year and 27.2 (95% CI: 26.8, 27.1) at 3-year, and a gradual decrease subsequently. The BMI trajectory was significantly lower in HD patients who died than those who survived, although this pattern was not observed in PD patients. Compared to the reference time-varying BMI category of 25.1–28 kg/m^2^, the mortality risks of both HD and PD patients were greater in all categories of time-varying BMI <25 kg/m^2^. The mortality risks were significantly lower in all categories of time-varying BMI >28.1 kg/m^2^ among HD patients, but only in the category 28.1–31 kg/m^2^ among PD patients.

**Conclusions:**

BMI changed over time in a non-linear fashion in incident dialysis patients. Time-varying measures of BMI were significantly associated with mortality risk in both HD and PD patients.

## Introduction

Nearly two-thirds of the adult population in developed countries is either overweight or obese, defined as body-mass index (BMI) ≥25 kg/m^2^ [1], [2]. High BMI is a risk factor for end-stage kidney disease (ESKD) [3]. Patients with ESKD receiving dialysis are no exception to the obesity epidemic [4]. In the USA, the mean BMI of incident ESKD patients has increased from 25.7 kg/m^2^ in 1995 to 27.5 kg/m^2^ in 2002 [4], and to 28.9 kg/m^2^ in 2007–2009 [5]. In the general population, obesity is associated with increased risks of all-cause and cardiovascular mortality [6], [7]. Therefore, weight loss therapy is recommended for people with BMI ≥30 kg/m^2^ or BMI between 25 and 29.9 kg/m^2^ with ≥2 absolute cardiovascular risk factors [8], [9]. However, there are currently no such treatment guidelines for ESKD patients receiving chronic dialysis for two main reasons. First, malnutrition is extremely common in dialysis patients and is a major risk factor of mortality [10]. Second, high BMI is associated with a survival advantage in dialysis patients [11]. The paradox of better survival with higher BMI has been well described in ESKD patients receiving hemodialysis (HD) [11]–[15]. The association between obesity and survival is less clear in ESKD patients receiving peritoneal dialysis (PD), with studies variably showing better [16], [17], comparable [11], [18], [19], or worse [14], [20] survival. Due to this conflicting evidence, except for severely obese patients who are waiting for kidney transplant surgery, it is largely unclear whether to recommend weight loss therapy to dialysis patients who are either overweight or obese. The studies evaluating BMI and mortality are based on a BMI value at baseline or at the commencement of dialysis therapy. Studies using follow-up measures of BMI (time-varying) are sparse in both HD and PD patients [12], [21]–[27]. Therefore, we examined the BMI trajectory and its association with all-cause mortality in incident ESKD patients on both HD and PD using data from the Australia and New Zealand Dialysis and Transplant (ANZDATA) Registry.

## Methods

This longitudinal cohort study was undertaken in accordance with the STROBE (Strengthening the Reporting of Observational Studies in Epidemiology) Statement [28]. The study included 20524 patients from the ANZDATA Registry who commenced chronic dialysis therapy between January 1, 2001 and December 31, 2008, comprising all incident patients with ESKD in Australia and New Zealand during that time. Patients were excluded if they were younger than 18 years at the time of renal replacement therapy (RRT) commencement or did not complete at least 6 months on dialysis since commencement of RRT (due to death or recovery of kidney function or transplantation or follow-up<6 months) or underwent pre-emptive kidney transplantation. Patients with <2 available follow-up measurements of weight or typographical error (for example, 9 kg instead of 90 kg) were also excluded. Final analysis included 17022 patients (Figure S1 in S1 Information).

The ANZDATA Registry collects information on all patients receiving RRT from all renal units throughout Australia and New Zealand, and has been extensively used for clinical epidemiological studies [29], [30]. The data collection is conducted in accordance with the Australian Commonwealth Privacy Act and associated state legislation governing health data collection; and individual patient consent is not required for the registry data. The anonymity of patient information is maintained by the coding of data during compilation; only anonymized data is released by the registry to the researchers. The ANZDATA Registry has approved this study and the submission of this manuscript. The data were collected every 6 months until March 31, 2004 (survey dates 31^st^ March and 30^th^ September) and annually since December 31, 2004 (survey date 31^st^ December). The structure of the ANZDATA Registry, the methods of data collection and validation are described in detail on its website (http://www.anzdata.org.au). In summary, the collection is complete from the first RRT procedure in Australia and New Zealand in 1963 and includes all patients from all renal units in both countries. The data collected included demographic details, underlying cause of ESKD, a limited range of comorbidities (the presence of coronary artery disease, peripheral vascular disease, cerebrovascular disease, chronic lung disease, diabetes mellitus, hypertension, and cigarette smoking), late nephrologist referral (<3 months before dialysis start), serum creatinine at dialysis start, the type and dates of each dialysis episode, details about kidney transplantation, and measurements of height and dry weight.

RRT modality was classified into HD (including hospital-, satellite-, and home-based HD), PD (including continuous ambulatory PD and automated PD), and renal transplantation. Dialysis modality was assigned as modality used at 90 days after commencement of RRT. BMI was calculated from the quotient of the weight and the square of the height at the commencement of RRT. The values of BMI were divided into 9 categories (≤19, 19.1–22, 22.1–25, 25.1–28, 28.1–31, 31.1–34, 34.1–37, 37.1–40 and >40.1 kg/m^2^).

### Statistical analysis

The basic statistics on study variables were expressed as number (%), mean (SD) or median (IQR), as appropriate. The distributions of categorical and continuous study variables by two dialysis modalities were compared using χ2 test and non-parametric Mann-Whitney U test, respectively.

The BMI trajectory was constructed by plotting the mean (95% CI) of baseline and follow-up measures of BMI against time points. For the BMI trajectory analysis, data at the following time points during follow-up were utilized: baseline; 3-, 6-, 9-months; 1-, 1.5-, 2-, 2.5-, 3-, 4-, 5-, 6-, 7- and 8-years. These time periods were rounded to the nearest survey date of the ANZDATA Registry data collection. Complete case analysis with follow-up measures of BMI was conducted, and no missing data imputation was employed. Generalized Estimating Equation based multivariate regression models were used to compare the BMI trajectories. Multivariate Cox regression models were used to evaluate risk factors of all-cause mortality. To evaluate the association between follow-up BMI measures and all-cause mortality, BMI was used as a time-varying covariate. Other covariates used in the multivariate regression models were: gender, age, dialysis modality, ethnicity, cause of ESKD, smoking status, diabetes mellitus, chronic lung disease, coronary artery disease, cerebrovascular disease, peripheral vascular disease, and late referral. Due to estimation convergence problems, the time-varying dialysis modality could not be used, and a fixed dialysis modality variable (modality used at 90 days after commencement of RRT) was used as a covariate instead. Robust standard errors of the hazard ratios (HR) were estimated after adjusting for the effects of study centres as random effects. The “proportional hazard” assumption was tested using standard likelihood ratio test. Survival time was calculated from the date of commencement of RRT to the date of event or censoring. Survival analyses were censored for kidney transplantation, loss of follow-up, recovery of renal function or December 31, 2008.

## Results

### Patient characteristics

Baseline patient characteristics are described in Table 1. Compared to those on PD, HD patients were more likely to be males, Caucasians, Aboriginals or Torres Strait Islanders, referred late to nephrologist before commencement of dialysis and to have comorbid conditions, including diabetes mellitus, chronic lung disease and coronary artery disease (Table 1). PD patients had lower average weight and BMI values, and were less likely to have BMI >34 kg/m^2^ compared to HD patients. Distributions of BMI groups according to the ethnicity and dialysis modality are described in Table S1 in S1 Information.

**Table 1:** Baseline patient characteristics.

Characteristic	All	Hemodialysis	Peritoneal dialysis
n*	17,022	10,860 (63.8)	6,162 (36.2)
Men*	10,249 (60.2)	6,793 (62.6)	3,456 (56.1)
Age (years)#	60.4 (15)	60.6 (15.1)	60.3 (14.7)
Age categories*			
18 to 45 years	2,820 (16.6)	1,782 (16.4)	1,038 (16.9)
45 to 65 years	6,791 (39.9)	4,330 (39.9)	2,461 (39.9)
>65 years	7,411 (43.5)	4,748 (43.7)	2,663 (43.2)
Ethnicity*			
Caucasians	12,324 (72.4)	8,030 (73.9)	4,294 (69.7)
Aboriginal or Torres Strait Islander	1,397 (8.2)	1,046 (9.6)	351 (5.7)
Maori or Pacific Islander	1,840 (10.8)	1,063 (9.8)	777 (12.6)
Asian	785 (4.6)	370 (3.4)	415 (6.7)
Other	676 (4)	351 (3.2)	325 (5.3)
Primary cause of end-stage kidney disease*			
Chronic glomerulopathy	4,155 (24.4)	2,591 (23.9)	1,564 (25.4)
Diabetic nephropathy	5,729 (33.7)	3,627 (33.4)	2,102 (34.1)
Renovascular disease	2,966 (17.4)	1,862 (17.2)	1,104 (17.9)
Polycystic disease	1,033 (6.1)	688 (6.3)	345 (5.6)
Reflux or obstructive nephropathy	827 (4.9)	542 (5)	285 (4.6)
Other	1,256 (7.4)	871 (8)	385 (6.3)
Unknown	1,056 (6.2)	679 (6.3)	377 (6.1)
Smoking status* †			
Non-smoker	7,688 (45.2)	4,807 (44.3)	2,881 (46.8)
Former smoker	6,965 (40.9)	4,533 (41.8)	2,432 (39.5)
Current smoker	2,365 (13.9)	1,517 (14)	8,48 (13.8)
Weight (kg)#	77.4 (19.9)	79.5 (21.4)	73.6 (16.5)
Body mass index (kg/m^2^)#	27.6 (6.4)	28.2 (6.9)	26.6 (5.3)
Body mass index category*			
≤19 kg/m^2^	750 (4.4)	480 (4.4)	270 (4.4)
19–22 kg/m^2^	2,182 (12.8)	1,295 (11.9)	887 (14.4)
22–25 kg/m^2^	3,563 (20.9)	2,161 (19.9)	1,402 (22.8)
25–28 kg/m^2^	3,615 (21.2)	2,180 (20.1)	1,435 (23.3)
28–31 kg/m^2^	2,711 (15.9)	1,707 (15.7)	1,004 (16.3)
31–34 kg/m^2^	1,795 (10.6)	1,179 (10.9)	616 (10)
34–37 kg/m^2^	1,008 (5.9)	712 (6.6)	296 (4.8)
37–40 kg/m^2^	615 (3.6)	458 4.2)	157 (2.6)
>40 kg/m^2^	783 (4.6)	688 (6.3)	95 (1.5)
Comorbid conditions*			
Diabetes mellitus	7,382 (43.4)	4,773 (44)	2,609 (42.3)
Chronic lung disease	2,679 (15.7)	1,775 (16.3)	904 (14.7)
Coronary artery disease	6,807 (40)	4,470 (41.2)	2,337 (37.9)
Cerebrovascular disease	2,492 (14.6)	1,583 (14.6)	909 (14.8)
Peripheral vascular disease	4,278 (25.1)	2,762 (25.4)	1,516 (24.6)
Late referral‡ †	4,057 (23.8)	2,757 (25.4)	1,300 (21.1)

*n (%).

# mean (standard deviation).

† Some missing data.

‡ Late referral defined as referral to nephrologist >3 months before dialysis start.

### BMI trajectory in incident dialysis patients

**Figure 1:**
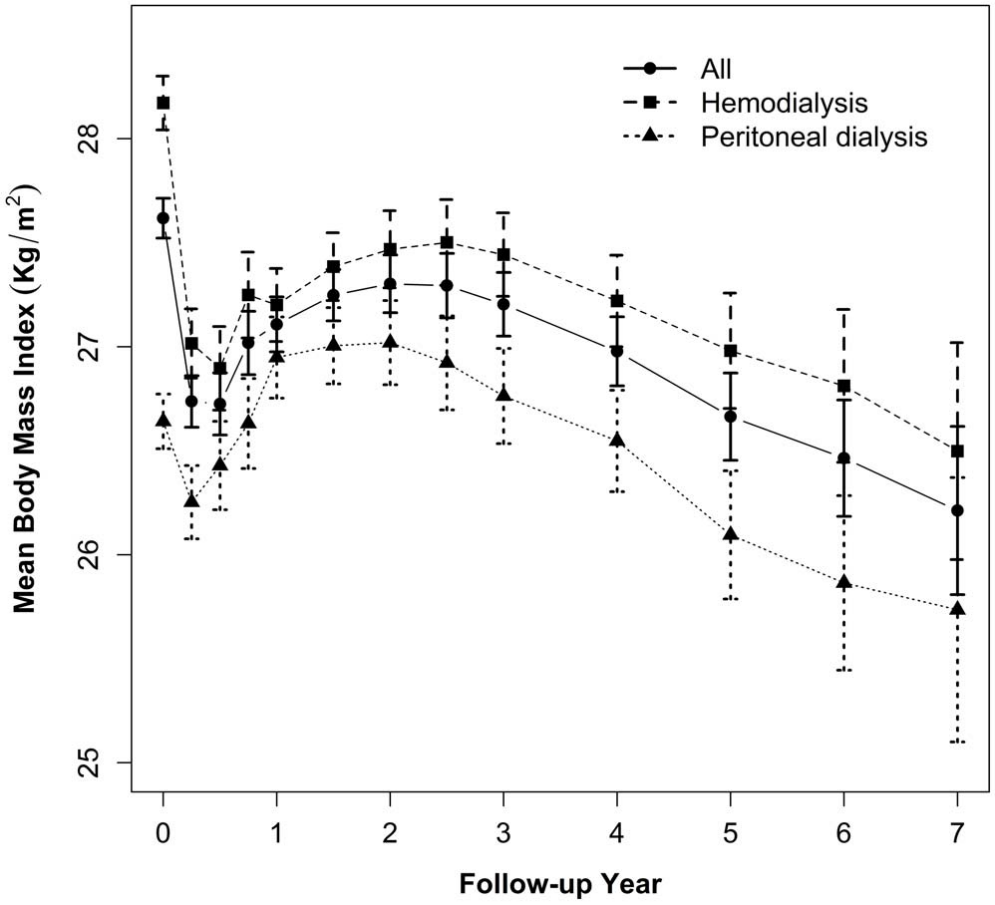
Body Mass Index Trajectories in All Incident Dialysis Patients and According to Dialysis Modality.

The median follow-up time was 2.3 years (range: 6 months to 8 years) and the median number of weight measurements per patient was 4 with a range from 2 to 13 (See Table S2 in S1 Information). BMI changed over time in a non-linear fashion (Fig. 1). Mean BMI initially decreased significantly from 27.6 (95% confidence interval [CI]: 27.5, 27.7) kg/m^2^ at baseline to 26.7 (95% CI: 26.6, 26.9) kg/m^2^ at 3 months, then increased to 27.1 (95% CI: 27, 27.2) kg/m^2^ at 1 year. It then remained stable over the next 2 years with a mean BMI of 27.2 (95% CI: 26.8, 27.1) kg/m^2^ at 3 years. Thereafter, mean BMI decreased gradually. The average baseline BMI in the HD group was significantly higher by 1.6 kg/m^2^ (95% CI: 1.4, 1.8, *P*<0.001) than the PD group, and the BMI trajectory in the HD group remained at a significantly higher level than the PD group throughout the follow-up period (Fig. 1). Despite a significant difference in the baseline BMI, the patterns of non-linear change in BMI were similar in both groups. In PD patients, the average BMI at 1 year of follow-up was 0.4 kg/m^2^ (95% CI: 0.2, 0.6) higher than the baseline level. However, in HD patients, follow-up BMI values were consistently below the baseline level.

### Baseline BMI and all-cause mortality

A total of 5,971 (35%) patients died during follow-up (3,671[34%] in the HD group vs. 2,300[37%] in the PD group) with a death rate of 12.7 (95% CI: 12.4, 13.1) per 100 person-years. The death rates per 100 person-years in the HD and PD groups were 12.2 (95% CI: 11.8, 12.6) and 13.7 (95% CI: 13.1, 14.3), respectively (See Table S3 in S1 Information for causes of death).

**Figure 2:**
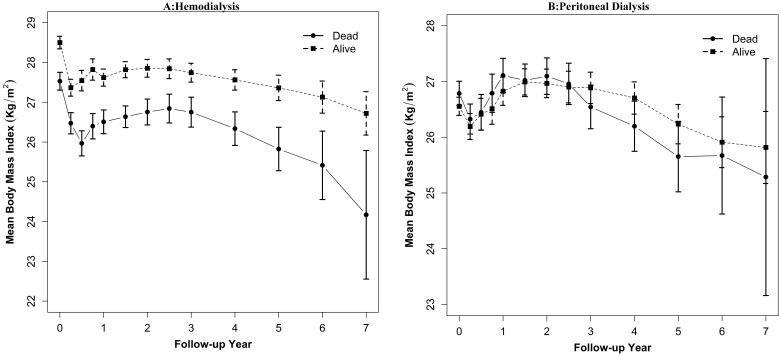
Body Mass Index Trajectory and Mortality in Incident Dialysis Patients: A, Hemodialysis; B, Peritoneal dialysis.

The baseline BMI was higher by 0.6 kg/m^2^ (95% CI: 0.4, 0.8, *P*<0.001) in patients who remained alive than those who died. At baseline, the surviving cohort had higher BMI by 1 kg/m^2^ (*P*<0.01) in HD patients (Fig. 2, panel A). However, the baseline BMI was not different by mortality status in the PD group (Fig. 2, panel B). Compared to the reference BMI category of 25–28 kg/m^2^ at baseline, all categories of BMI <25 kg/m^2^ were associated with increased mortality risk for all dialysis patients (Table 2). However, the risk estimates were not consistent between the HD and PD groups. Higher baseline BMI was associated with significantly lower mortality risk for HD patients with BMI between 28 and 37 kg/m^2^. The mortality risk was significantly higher in the PD group with BMI 34–37 kg/m^2^.

**Table 2:** The association between baseline BMI and all-cause mortality.

	All patients			HD patients			PD patients		
BMI (kg/m^2^)	HR	95%CI	*P*	HR	95%CI	*P*	HR	95%CI	*P*
≤19	1.55	1.37, 1.77	<0.001	1.62	1.37, 1.92	<0.001	1.43	1.15, 1.78	<0.01
19 to 22	1.14	1.05, 1.23	<0.01	1.16	1.03, 1.30	0.02	1.08	0.96, 1.22	0.2
22 to 25	1.12	1.04, 1.20	0.003	1.07	0.98, 1.17	0.1	1.17	1.03, 1.32	0.02
25 to 28	Reference			Reference			Reference		
28 to 31	0.96	0.89, 1.03	0.3	0.90	0.81, 0.99	0.03	1.05	0.94, 1.18	0.4
31 to 34	0.92	0.84, 1.01	0.08	0.82	0.74, 0.90	<0.001	1.11	0.96, 1.28	0.2
34 to 37	0.95	0.86, 10.4	0.3	0.78	0.68, 0.89	<0.001	1.33	1.12, 1.57	<0.01
37 to 40	0.97	0.82, 1.14	0.7	0.90	0.73, 1.11	0.3	1.05	0.76, 1.46	0.8
>40	0.96	0.83, 1.11	0.6	0.89	0.77, 1.03	0.1	1.11	0.77, 1.61	0.6

### Time-varying BMI and all-cause mortality

The BMI trajectory was significantly higher on average by 1.22 kg/m^2^ (95% CI: 1.2, 1.3) in those who remained alive compared to those who died. The BMI trajectories in HD and PD patients who survived were higher on average by 1.5 kg/m^2^ (95% CI: 1.4, 1.6) and 0.5 kg/m^2^ (95% CI: 0, 0.6), respectively, than those who died. The adjusted risks of mortality associated with different categories of time-varying BMI for all patients and separately for dialysis modality are presented in Table 3. Compared to the reference time-varying BMI category of 25–28 kg/m^2^, there was significantly increased mortality risk in all categories with time-varying BMI<25 kg/m^2^. There was also a decreased risk of mortality for all categories of time-varying BMI >28 kg/m^2^, except for 37–40 kg/m^2^. When analyzed separately for dialysis modalities, increased mortality risks with all categories of time-varying BMI <25 kg/m^2^ were evident in both HD and PD groups. Decreased mortality risk was evident with all categories of time-varying BMI >28 kg/m^2^ in the HD group. However, decreased mortality risk in the PD group was observed only up to the time-varying BMI category of 28–31 kg/m^2^. In the PD group, there were no significant differences in the risk estimates for higher time-varying BMI categories (up to 37 kg/m^2^); although a trend of increasing mortality risk was observed for the time-varying BMI category 37–40 kg/m^2^. The risk estimates for other risk factors from the multivariate Cox regression models are presented in Table S4 in S1 Information.

**Table 3:** The association between time-varying BMI and all-cause mortality.

	All patients			HD patients			PD patients		
BMI (kg/m^2^)	HR	95%CI	*P*	HR	95%CI	*P*	HR	95%CI	*P*
≤19	3.30	2.91, 3.74	<0.001	3.72	3.27, 4.24	<0.001	2.56	2.10, 3.12	<0.001
19 to 22	1.70	1.53, 1.89	<0.001	1.74	1.54, 1.98	<0.001	1.63	1.41, 1.89	<0.001
22 to 25	1.23	1.13, 1.34	<0.001	1.25	1.11, 1.40	<0.001	1.22	1.06, 1.40	<0.01
25 to 28	Reference			Reference			Reference		
28 to 31	0.80	0.73, 0.88	<0.001	0.80	0.71, 0.91	0.001	0.80	0.69, 0.94	<0.01
31 to 34	0.82	0.74, 0.90	<0.001	0.72	0.65, 0.81	<0.001	0.96	0.80, 1.14	0.6
34 to 37	0.82	0.71, 0.94	<0.01	0.69	0.59, 0.81	<0.001	1.06	0.83, 1.34	0.7
37 to 40	0.94	0.81, 1.09	0.4	0.76	0.61, 0.95	0.02	1.30	1.05, 1.62	0.02
>40	0.76	0.64, 0.90	<0.01	0.74	0.63, 0.87	<0.001	0.72	0.51, 1.01	0.06

## Discussion

This large registry study showed that BMI changed over time in a non-linear fashion in incident dialysis patients. Although the patterns of non-linear change in BMI were similar in HD and PD groups, the average BMI in PD patients at 1 year of follow-up was slightly higher than the baseline level. Time-varying measures of BMI were significantly associated with mortality risk in both HD and PD patients.

High BMI is a risk factor for ESKD and the prevalence of obesity is increasing among ESKD patients needing dialysis [3]. Most of the studies evaluating the association between BMI and mortality have used a single BMI value at baseline. Few studies have assessed follow-up measures of BMI or weight [12], [21]–[27]. Using data from a large dialysis provider, Kalantar-Zadeh and colleagues reported a significantly increased mortality risk in HD patients with time-varying BMI values below the reference category of 23 to 25 kg/m^2^ and a decreased mortality risk with high BMI categories, including very high BMI values of >45 kg/m^2^ [12], [21], [22], [25]. Kotanko and colleagues observed a marked decrease in body weight in 3 months preceding death in HD patients [26]. Using serum creatinine as a surrogate for muscle mass, decline in muscle mass was a stronger predictor of mortality than weight loss [25]. The patient characteristics from these studies were different to those from our study. First, the previous studies included prevalent dialysis patients that may have led to the introduction of selective survival bias [31]. Second, the ethnic compositions of the cohort were different as our study predominantly included Caucasian patients. Third, except one [27], all studies included patients receiving HD only. Previously reported analyses of the effect of intra-individual change in (gain or loss of) BMI or weight over time on mortality could have been affected by regression to the mean [32] and an incorrect assumption of linear and unidirectional change of weight.

A key strength of our study was the inclusion of all incident adult ESKD patients receiving both HD and PD. The initial decrease in BMI in the first year of starting dialysis could be due to the excess burden of illness that these patients experience at the time of reaching ESKD necessitating dialysis. Indeed, mortality is highest in the first year of starting dialysis with reported rates ranging from 17.5% to 25% [5], [33]. Due to the absence of data on the assessment of volume status, we could not differentiate between weight loss due to fluid removal and true weight loss. Subsequent weight gain could be due to improved appetite and nutrition once patients are stabilised on dialysis therapy. As with HD patients, there was an increased risk of mortality in PD patients with lower time-varying BMI. In contrast, the survival benefit associated with high BMI in PD patients was limited to the time-varying BMI category of 28 to 31 kg/m^2^. There were no significant differences in the risk estimates for time-varying BMI up to 37 kg/m^2^, although a trend of increasing mortality risk was observed that achieved statistical significance for the time-varying BMI category of 37 to 40 kg/m^2^. This discrepancy in mortality risks between the two dialysis modalities at higher BMI values may be due to differential development of abdominal obesity, which is in turn associated with increased mortality in ESKD patients [34]. Visceral fat mass increases by 11–23% within 1 year of initiating PD, probably due to the metabolic consequences of intraperitoneal administration of glucose-containing PD solutions [35]–[37]. In contrast, increases in visceral fat mass are generally not observed in HD patients [38], [39]. Importantly, our study demonstrated that the average BMI at 1 year of follow-up was higher than the baseline value in PD patients, but lower in HD patients. We did not observe an increased mortality risk in PD patients with time-varying BMI >40 kg/m^2^, possibly due to small patient numbers (95 patients).

A major limitation of our study was that BMI does not distinguish fat mass from lean mass, especially in patients with muscle wasting [40]. Furthermore, BMI does not reflect body-fat distribution. Given that fat distribution varies substantially across various ethnic backgrounds at the same level of BMI, BMI is not an ideal surrogate of fat mass [41]. The ANZDATA Registry does not collect data on other anthropometric measures, body composition, laboratory indices of nutrition and inflammation, hospitalizations or cardiovascular events. The accuracy of reported data on dry-weight could not be validated as data on the assessment of volume status are not collected. One common limitation with survival regression models incorporating time-varying covariates is the difficulty to identify the non-linearity of the time-varying covariates. However, given the observed BMI trajectory, the hazard estimates obtained through the weighted Cox regression models should be sufficiently robust. Other limitations of our study include adjustment for a limited number of baseline variables and variable frequency of weight reporting. Patients with less than 6 months follow-up on dialysis were excluded, potentially leading to bias. Since our study predominantly included Caucasian patients, the results of this study may not be generalizable to patient populations with different ethnic compositions.

Considering the obesity epidemic in ESKD, the results of our study have important clinical and research implications, despite its limitations. The finding of a non-linear change in BMI, especially in the 1^st^ year of starting dialysis, suggests that particular attention should be paid by clinicians to optimising nutritional intake in this early period in the hope of preventing early weight loss and heightened early mortality. Prospective studies are required to understand the dynamic association between BMI and abdominal obesity in dialysis patients and its variation according to dialysis modality. Randomised clinical trials are required to study the effect of nutritional interventions on BMI and important clinical outcomes, such as quality of life and mortality. Finally, research evaluating the effects of glucose-sparing PD regimens on visceral fat mass and patient outcomes is needed.

In conclusion, BMI changed in a non-linear fashion in incident dialysis patients. Time-varying measures of BMI were significantly associated with all-cause mortality risk. Lower time-varying BMI categories were associated with increased mortality risk in both HD and PD patients. Higher time-varying BMI categories were associated with decreased mortality in HD patients, but not in PD patients.

## Supporting Information

S1 Information **Supporting tables and figure.** Table S1. Distributions of BMI groups according to ethnicity and dialysis modality. Table S2. Number of weight measurements per patient. Table S3. Causes of death. Table S4: Hazard ratio (95% CI) for mortality associated with individual covariates from the multivariate Cox-regression model with time-varying BMI as covariate. Figure S1. Flow diagram describing patient selection in the study.
(DOCX)
