# Supplementary material for: The Association between Body Mass Index and Mortality in Incident Dialysis Patients
Source: PLoS One. 2014 Dec 16;9(12):e114897. doi: 10.1371/journal.pone.0114897 (PMC4267775; doi:10.1371/journal.pone.0114897)
Supplement: S1 Information — Supporting tables and figure. Table S1. Distributions of BMI groups according to ethnicity and dialysis modality. Table S2. Number of weight measurements per patient. Table S3. Causes of death. Table S4: Hazard ratio (95% CI) for mortality associated with individual covariates from the multivariate Cox-regression model with time-varying BMI as covariate. Figure S1. Flow diagram describing patient selection in the study. (DOCX) [file pone.0114897.s001.docx]

**Table S1. Distributions of BMI groups according to ethnicity and dialysis modality**

| **Body mass index category** | **Caucasians** | **Aboriginal or Torres Strait Islander** | **Maori or Pacific Islander** | **Asian** | **Other** |
| --- | --- | --- | --- | --- | --- |
| ***Hemodialysis*** | | | | | |
| ≤19 kg/m^2^ | 362 (4.5) | 53 (5.1) | 2 (<1) | 41 (11.1) | 22 (6.3) |
| 19 – 22 kg/m^2^ | 979 (12.2) | 142 (13.6) | 32 (3) | 81 (21.9) | 61 (17.4) |
| 22 – 25 kg/m^2^ | 1,674 (20.9) | 188 (18) | 96 (9) | 122 (33) | 81 (23.1) |
| 25 – 28 kg/m^2^ | 1,691 (21.1) | 196 (18.7) | 137 (12.9) | 65 (17.6) | 91 (25.9) |
| 28 – 31 kg/m^2^ | 1,311 (16.3) | 157 (15) | 173 (16.3) | 30 (8.1) | 36 (10.3) |
| 31 – 34 kg/m^2^ | 834 (10.4) | 113 (10.8) | 181 (17) | 18 (4.9) | 33 (9.4) |
| 34 – 37 kg/m^2^ | 468 (5.8) | 80 (7.7) | 146 (13.7) | 6 (2) | 12 (3) |
| 37 – 40 kg/m^2^ | 307 (3.8) | 54 (5.2) | 90 (8.5) | 3 (1) | 4 (1) |
| >40 kg/m^2^ | 404 (5) | 63 (6) | 206 (19.4) | 4 (1) | 11 (3.1) |
| ***Peritoneal dialysis*** | | | | | |
| ≤19 kg/m^2^ | 196 (4.6) | 15 (4.3) | 9 (1.2) | 29 (7) | 21 (6.5) |
| 19 – 22 kg/m^2^ | 619 (14.4) | 54 (15.4) | 28 (3.6) | 114 (27.5) | 72 (22.2) |
| 22 – 25 kg/m^2^ | 1,017 (23.7) | 66 (18.8) | 112 (14.4) | 122 (29.4) | 85 (26.2) |
| 25 – 28 kg/m^2^ | 1,019 (23.7) | 77 (21.9) | 181 (23.3) | 83 (20) | 75 (23.1) |
| 28 – 31 kg/m^2^ | 708 (16.5) | 53 (15.1) | 157 (20.2) | 38 (9.2) | 48 (14.8) |
| 31 – 34 kg/m^2^ | 417 (9.7) | 42 (12) | 129 (16.6) | 15 (3.6) | 13 (4) |
| 34 – 37 kg/m^2^ | 169 (3.9) | 26 (7.4) | 84 (10.8) | 9 (2.2) | 8 (2.5) |
| 37 – 40 kg/m^2^ | 95 (2.2) | 11 (3.1) | 44 (5.7) | 5 (1.2) | 2 (1) |
| >40 kg/m^2^ | 54 (1.3) | 7 (2) | 33 (4.3) | 0 (0) | 1 (<1) |

n (%)

**Table S2. Number of weight measurements per patient**

| **Number of weight measurements per patients** | **All patients** | **HD** | **PD** |
| --- | --- | --- | --- |
| 2 | 1,544 | 983 | 561 |
| 3 | 3,980 | 2572 | 1408 |
| 4 | 3,547 | 2222 | 1325 |
| 5 | 2,498 | 1650 | 848 |
| 6 | 1,595 | 1007 | 588 |
| 7 | 1,115 | 699 | 416 |
| 8 | 859 | 549 | 310 |
| 9 | 680 | 415 | 265 |
| 10 | 488 | 303 | 185 |
| 11 | 351 | 221 | 130 |
| 12 | 263 | 175 | 88 |
| 13 | 102 | 64 | 38 |

**Table S3. Causes of death**

| **Cause of death [n (%)]** | **All** | **HD** | **PD** |
| --- | --- | --- | --- |
| Cardiac | 2,283 (38.8) | 1,361 (37.1) | 922 (40.1) |
| Vascular | 535 (9) | 330 (9) | 205 (8.9) |
| Infection | 691 (11.6) | 382 (10.4) | 309 (13.4) |
| Social | 1,797 (30.1) | 1,168 (31.8) | 629 (27.4) |
| Miscellaneous | 665 (11.1) | 430 (11.7) | 235 (10.5) |
| **Total deaths** | **5,971** | **3,671** | **2,300** |

**Table S4: Hazard ratio (95% CI) for mortality associated with individual covariates from the multivariate Cox-regression model with time-varying BMI as covariate**

|  | **All patients** | | | **HD patients** | | | **PD patients** | | |
| --- | --- | --- | --- | --- | --- | --- | --- | --- | --- |
| **Variable** | **HR** | **95%CI** | **P** | **HR** | **95%CI** | **P** | **HR** | **95%CI** | **P** |
| Women | 1.02 | 0.96, 1.09 | 0.54 | 0.98 | 0.91, 1.05 | 0.54 | 1.11 | 1.01, 1.21 | 0.03 |
| Age (per 1 year increase) | 1.03 | 1.03, 1.04 | <0.001 | 1.03 | 1.03, 1.03 | <0.001 | 1.04 | 1.03, 1.04 | <0.001 |
| HD vs. PD | 0.81 | 0.76, 0.87 | <0.001 | Not applicable | | | | | |
| Ethnicity | | | | | | | | | |
| Caucasians | Reference | | | Reference | | | Reference | | |
| Aboriginal or Torres Strait Islander | 1.00 | 0.88, 1,13 | 0.94 | 0.92 | 0.78, 1.08 | 0.29 | 1.17 | 1.01, 1.34 | 0.03 |
| Maori or Pacific Islander | 1.35 | 1.14, 1.61 | <0.01 | 1.37 | 1.09, 1.73 | <0.01 | 1.32 | 1.10, 1.58 | <0.01 |
| Asian | 0.52 | 0.43, 0.63 | <0.001 | 0.49 | 0.39, 0.62 | <0.001 | 0.55 | 0.42, 0.72 | <0.001 |
| Other | 0.55 | 0.46, 0.65 | <0.001 | 0.48 | 0.36, 0.64 | <0.001 | 0.65 | 0.55, 0.76 | <0.001 |
| Primary cause of ESKD | | | | | | | | | |
| Chronic glomerulopathy | Reference | | | Reference | | | Reference | | |
| Diabetic nephropathy | 1.33 | 1.20, 1.47 | <0.001 | 1.31 | 1.15, 1.49 | <0.001 | 1.37 | 1.16, 1.61 | <0.001 |
| Renovascular disease | 1.28 | 1.15, 1.42 | <0.001 | 1.31 | 1.14, 1.51 | <0.001 | 1.21 | 1.05, 1.39 | <0.01 |
| Polycystic disease | 0.72 | 0.61, 0.85 | <0.001 | 0.73 | 0.59, 0.89 | <0.01 | 0.69 | 0.54, 0.89 | <0.01 |
| Reflux or obstructive nephropathy | 1.20 | 1.03, 1.38 | 0.02 | 1.29 | 1.07, 1.54 | <0.01 | 0.97 | 0.78, 1.21 | 0.8 |
| Other | 1.98 | 1.75, 2.24 | <0.001 | 2.12 | 1.82, 2.46 | <0.001 | 1.70 | 1.36, 2.12 | <0.001 |
| Unknown | 1.37 | 1.20, 1.55 | <0.001 | 1.36 | 1.17, 1.59 | <0.001 | 1.34 | 1.10, 1.63 | <0.01 |
| Smoking status | | | | | | | | | |
| Non-smoker | Reference | | | Reference | | | Reference | | |
| Former smoker | 1.04 | 0.97, 1.11 | 0.24 | 1.04 | 0.96, 1.12 | 0.33 | 1.06 | 0.96, 1.16 | 0.27 |
| Current smoker | 1.09 | 0.99, 1.20 | 0.07 | 1.03 | 0.91, 1.17 | 0.63 | 1.22 | 1.07, 1.38 | <0.01 |
| Comorbid conditions | | | | | | | | | |
| Diabetes mellitus | 1.46 | 1.34, 1.60 | <0.001 | 1.57 | 1.43, 1.73 | <0.001 | 1.31 | 1.12, 1.52 | <0.01 |
| Chronic lung disease | 1.22 | 1.14, 1.31 | <0.001 | 1.24 | 1.14, 1.34 | <0.001 | 1.19 | 1.07, 1.33 | <0.01 |
| Coronary artery disease | 1.32 | 1.24, 1.41 | <0.001 | 1.31 | 1.20, 1.43 | <0.001 | 1.35 | 1.21, 1.49 | <0.001 |
| Cerebrovascular disease | 1.16 | 1.09, 1.25 | <0.001 | 1.13 | 1.03, 1.25 | <0.01 | 1.22 | 1.10, 1.34 | <0.001 |
| Peripheral vascular disease | 1.19 | 1.10, 1.28 | <0.001 | 1.11 | 1.02, 1.21 | 0.02 | 1.33 | 1.20, 1.47 | <0.001 |
| Late referral | 1.12 | 1.06, 1.19 | <0.001 | 1.11 | 1.02, 1.20 | 0.02 | 1.15 | 1.04, 1.26 | <0.01 |

**Figure S1.** Flow diagram describing patient selection in the study
